# Brain oscillatory activity in adolescent idiopathic scoliosis

**DOI:** 10.1038/s41598-022-19449-1

**Published:** 2022-10-14

**Authors:** Emanuela Formaggio, Margherita Bertuccelli, Maria Rubega, Roberto Di Marco, Francesca Cantele, Federica Gottardello, Michela De Giuseppe, Stefano Masiero

**Affiliations:** 1 Department of Neuroscience, Section of Rehabilitation, University of Padova, Via Giustiniani 3, 35128 Padova, Italy; 2 Padova Neuroscience Center, University of Padova, Via Orus 2/B, 35129 Padova, Italy; 3 Present Address: Department of Computer Science, University of Verona, Strada le Grazie 15, 37134 Verona, Italy

**Keywords:** Biomedical engineering, Diagnostic markers

## Abstract

Pathophysiology of Adolescent Idiopathic Scoliosis (AIS) is not yet completely understood. This exploratory study aims to investigate two aspects neglected in clinical practice: a defective postural central nervous system control in AIS, and alterations of body schema due to scoliosis spinal deformities. We recorded EEG data and balance data in four different standing positions in 14 adolescents with AIS and in 14 controls. A re-adaptation of the Image Marking Procedure (IMP) assessed body schema alterations on the horizontal (Body Perception Indices (*BPIs*)) and vertical direction (interacromial and bisiliac axes inclinations). Our results revealed no differences in balance control between groups; higher EEG alpha relative power over sensorimotor areas ipsilateral to the side of the curve and a significant increase of theta relative power localized over the central areas in adolescents with AIS. The difference in *BPI* shoulder and *BPI* waist significantly differed between the two groups. The inclinations of the perceived interacromial axes in adolescents with AIS was opposite to the real inclination. Increased theta activity and alpha lateralization observed may be a compensatory strategy to overcome sensorimotor dysfunction mirrored by altered body schema. Scoliosis onset might be preceded by sensorimotor control impairments that last during curve progression.

## Introduction

Adolescent Idiopathic Scoliosis (AIS) is a three-dimensional morphological spinal deformity, characterized by the deviation of the spine in the frontal plane ($> 10$ Cobb degrees) and the concurrent rotation of the affected vertebral bodies^1,2^. Epidemiological studies report an estimated prevalence of 1–3% among adolescents (10–16 years), with girls being more severely affected^3^.

Although being a widespread disease, studied for decades, the cause and the pathophysiology of AIS is not yet completely understood, but multi-factorial hypotheses have been proposed: genetic predisposition, hormonal dysfunctions^4^, defective postural control by the central nervous system (CNS)^5^, and alterations of body schema^6–8^. There is growing evidence of cortical involvement in AIS suggesting that AIS could be the expression of a sub-clinical nervous system disorder. Indeed, visuo-spatial perceptual impairments, body spatial orientation alterations, and sensory integration disorders have been described^9^. According to the neurodevelopmental theories of AIS, a temporal imbalance between the musculoskeletal maturation and the central re-adaptation of body schema would in turn result in improper trunk muscles response to compensate for scoliosis initiating process^9^. Body schema is defined as the internal, unconscious representation of our body boundaries and shape, constantly updated by sensory information (e.g., visual, proprioceptive, tactile, kinesthetic) and responsible for setting proper motor output^10^. Just a few studies tried to assess body schema in AIS clinical population, providing indirect indices of altered postural perception, and global lower awareness of the body^11^. However, a validated assessment instrument is still lacking. The postulated maturation delay of the CNS body schema^9^, could result from a peripheral impairment in sensory processing, a CNS multi-sensory integration impairment, and/or a motor output impairment.

Observations of impaired motor control were reported on brain imaging studies suggesting that an anomalous sensorimotor integration contributes to the cause of AIS, but there is uncertainty about the level of the central nervous system accounting for this dysfunction. Studies using structural magnetic resonance imaging (MRI), functional MRI (fMRI) and electroencephalography (EEG) revealed anomalies in the CNS: asymmetries in brainstem corticospinal bundles^12^, volumetric differences between adolescents with AIS and controls in brain regions functionally related to motor control and coordination^13^, and abnormal patterns in the motor network of adolescents during movement execution^14^.

Early studies investigating brain activity in scoliosis reported, for the first time, EEG paroxysmal activity at rest^15^—i.e., bilaterally synchronous activity spread over large areas of both hemispheres. An increase of the amplitude of the peak in alpha rhythm ([7.5–12.5] Hz) was found at central, frontal, parietal and occipital regions during standing posture indicating the need for increased cortical processing to maintain balance control in normal upright standing in adolescents with AIS compared to controls^16^. The increase in theta frequency power and suppression of alpha, beta and gamma powers were observed when proprioception was altered, during vibration applied over the tendons of the soleus/gastrocnemius and tibialis anterior of both ankles^17^. In this case the alpha desynchronization, reflecting the excitability of the sensorimotor cortex, provided indications that adolescents with AIS needed more cortical resources to process sensory information to control their body sway. A Transcranial Magnetic Stimulation (TMS) study reported lower intracortical inhibition, higher motor cortex excitability, and preserved spinal inhibitory circuits^18^, demonstrating evidence of central nervous system involvement in AIS.

The aims of this exploratory study are threefold: (1) to compare the EEG activity in adolescents with AIS and controls, to examine the brain oscillatory changes related to balance control; (2) to unveil possible alterations in the postural control mechanisms and (3) to investigate body schema alterations. Based on the literature background, we hypothesized that compared to controls, adolescents with AIS might show: (1) altered brain activation of the sensorimotor network; (2) larger sway on the frontal plane, as a result of a less efficient control of their balance on the plane with the larger scoliotic deformity; (3) altered body schema reflecting the altered activation of the sensorimotor network. Results may represent a valuable biomarker of AIS progression and offer novel therapeutic targets according to the identified pathophysiological patterns.

## Methods

### Participants

Fourteen adolescent girls with a confirmed diagnosis of AIS^1^ (age range 13–17 years; Cobb angle: 20$^{\circ }$–55$^{\circ }$; Risser sign: 0–4; no spine surgery) were recruited at the Adolescence Spine Diseases Diagnostic and Therapeutic Centre of the Padova University Hospital. Fourteen adolescent girls without AIS (age range 11–16 years; no spinal pathology or any known neurological or musculoskeletal disorders; no clinically relevant back hump), i.e., controls, were recruited at the Sports Medicine and at Physical Activities Unit–“ai Colli” Social Health Department of the Padova Hospital (Tables 1, 2). Controls (CTRL) were enrolled from a population of sporty females (involved in sport activities for less than 10 h per week) who visited the hospital for a certificate of good health, mandatory for any physical activity. A team of physicians with expertise in adolescent spine diseases collected the anamnestic information and the clinical data (Table 3). Parents gave their written informed consent to participate in the study. All methods were performed in accordance with relevant guidelines and regulations and in accordance with the Declaration of Helsinki.

**Table 1:** Adolescents with AIS’ descriptive characteristics.

Subject ID	Age (years)	Menarche (months)	Body Mass (kg)	Height (m)	BMI (kg/$\hbox {m}^2$)	Sport (type)	Sport (h/week)
1	17.1	25	53.5	1.69	18.73	Dance	3
2	13.7	12	52.0	1.62	19.94	–	–
3	13.9	19	44.0	1.61	17.08	Dance	1
4	14.1	20	62.0	1.75	20.24	–	–
5	15.7	42	66.0	1.64	24.69	Gymnastic	6
6	14.0	25	57.0	1.65	20.94	Swim	3
7	15.0	No	45,5	1.71	15.65	Volleyball	4
8	16.3	17	38.0	1.48	17.35	–	–
9	13.6	13	54.0	1.63	20.32	Dance	7
10	14.0	33	54.0	1.48	24.65	–	–
11	15.1	34	54.0	1.64	20.20	Dance	5
12	15.7	43	44.0	1.62	16.77	Atlethics	5
13	14.2	40	54.0	1.64	20.08	–	–
14	15.9	46	44.0	1.58	17.63	–	–

**Table 2:** Controls’ descriptive characteristics.

Subject ID	Age (years)	Menarche (months)	Body mass (kg)	Height (m)	BMI (kg/$\hbox {m}^2$)	Sport (type)	Sport (h/week)
1	14.7	43	84.5	1.6	33.64	Gymnastic	9
2	14.0	13	48.5	1.6	19.80	Gymnastic	10
3	14.0	24	48.5	1.6	18.83	–	–
4	11.6	1	50.0	1.5	20.95	Boxe	3
5	13.4	8	52.0	1.6	21.37	Volleyball	7
6	15.5	46	52.0	1.6	20.44	Atlethics	3
7	14.8	21	67.0	1.6	25.22	Gymnastic	8
8	15.5	36	45.0	1.6	17.47	Gymnastic	5
9	14.2	13	52.0	1.8	16.98	Handball	4,5
10	14.6	24	56.5	1.6	21.14	Gymnastic	9
11	13.7	4	50.0	1.7	17.30	Swim	10
12	13.6	26	49.0	1.6	20.26	Dance	5
13	16.6	65	70.5	1.7	24.39	Volleyball	7
14	14.9	36	50.0	1.6	20.16	Dance	5

**Table 3:** Anthropometric characteristic of adolescents with AIS.

Subject ID	Brace (months)	Cobb (°)	Curve site	Curve lateralization	Risser Sign (%)
1	48	20	Thoracic	Right	100
2	20	21	Thoracic	Right	80
3	30	38	Thoracic	Right	100
4	1	43	Thoracic	Right	75
5	19	21	Lumbar	Right	100
6	4	24	Thoracic	Right	40
7	19	25	Thoracolumbar	Right	90
8	7	55	Thoracic	Right	85
9	1	25	Lumbar	Right	10
10	65	22	Thoracic	Right	100
11	27	27	Lumbar	Left	60
12	1	24	Lumbar	Left	100
13	30	28	Lumbar	Left	85
14	41	28	Lumbar	Left	100

All the participants underwent a physical examination: trunk asymmetries, shoulders and hips inclination sides were registered. Bunnel’s scoliometer was used to measure the paravertebral humps in the forward bent position^19,20^. For adolescents with AIS only, we collected the last standing full-spine posterior-anterior radiological image recorded within the last three months prior participating in the study. Cobb angle method was used to measure curves entity^21^, while the apical vertebral rotation was recorded by means of the Perdriolle method^22^ either or Nash and Moe method^23^. The side and the anatomical site of the scoliosis convexity was collected (Table 3). All adolescents with AIS were treated using a full-time brace.

Prior to data collection, height and body mass were collected and handedness was assessed with the Oldfield Questionnaire^24^, with all participants being right-handed.

### Experimental paradigm

To answer our research questions and test the hypotheses we made, participants underwent a data collection session, which consisted of EEG, balance and motion recordings complemented with a procedure to assess the participants’ body schema. The instrumental data were acquired at once for each task and postural condition. Data synchronization was ensured, as balance and motion data were collected by the same motion capture system, which also served as a master to trigger the EEG acquisition. Participants were asked to stand upright on a force platform in two conditions:

- with their eyes: (1) open (*OE standing*), and (2) closed (*CE standing*) for 3 min;
- with their arms raised laterally to 90$^\circ$ and with: (3) eyes open (*OE arms up*), and (4) closed (*CE arms up*) for 1 min. This task has the twofold purpose of: (1) inducing a larger postural unbalance on the frontal plane, to further challenge the participants on that direction (i.e. where the larger scoliotic deformity displays); and (2) eliciting an asymmetrical activation of the spine muscles, which we expect to be unbalanced on adolescent with AIS, and to be reflected on an unbalanced activation of sensorimotor brain areas to counteract the effect of the scoliotic curve.

For each standing position, participants were instructed to keep their feet at their shoulder width, looking at a fixation cross ($\sim 3\,\hbox {m}$ in front of the participant) during the eyes open conditions (see Supplementary Materials).

Subsequently, participants underwent a re-adaptation of the Image Marking Procedure (IMP)^25^.

Adolescents with AIS performed the experiment without the brace removed two hours before the acquisition.

### EEG data

EEG signals (32-channels system; BrainAmp 32MRplus, BrainProducts GmbH, Munich, Germany) were acquired using an analogic anti-aliasing band pass-filter at 0.1–1000 Hz and converted from analog to digital using a sampling rate of 500 Hz. The reference was between Fz/Cz and ground anterior to Fz. The data were processed in Matlab R2018b (MathWorks, Natick, MA, Usa) using personalized scripts based on EEGLAB toolbox (http://www.sccn.ucsd.edu/eeglab)^26^. The EEG recordings were band-pass filtered from 1 to 30 Hz (the optimal Chebyshev finite impulse response filters were designed using Parks–McClellan algorithm, the order was customized to minimize the error in the pass and stop bands). Noisy channels were identified by visual inspection and interpolated using the nearest-neighbour spline method (average percentage of channels interpolated: 6.5%). Eyes movements and cardiac activity were removed using independent component analysis (FastICA algorithm implemented in EEGLAB) based on the waveform, topography and time course of the component (average percentage of components removed: 9.9%), and data were re-referenced to the average reference. Individual epochs containing non-stereotyped artifacts were also identified by visual inspection and removed from further analysis (average percentage of epochs removed: 7.4%, resulting in, on average, 43.1 EEG epochs of 2 s exploited for the following analysis).

The fast Fourier transform was applied to non–overlapping epochs of 2 s and then averaged across epochs. The recordings were Hanning windowed to control for spectral leakage. Power spectra were estimated for all frequencies between 1 and 30 Hz, then the relative power (%) was calculated by dividing the power of each frequency bands (delta [1–4] Hz, theta [4.5–7.5] Hz, alpha [8–12] Hz, and beta [13–30] Hz) with the total power of [1–30] Hz. The relative power was computed to reduce the inter-individual deviation associated with absolute power due to the inter-individual difference in skull and scalp conduction^27^. The frequency range was limited to [1–30] Hz considering that power spectral density shows a decrease in power with increasing frequency (1/f) and the presence of the power supply noise at 50 Hz.

A laterality index (LI), describing the contrast in amount of activation (i.e., relative power in alpha band) between the right and left hemisphere, was calculated during all the tasks according to:

1 $$\begin{aligned} \begin{aligned} LI = \frac{P_I-P_C}{P_I+P_C} \end{aligned} \end{aligned}$$

where $P_I$ is the average of power of central electrodes ipsilateral to the curve (C4, Cp2 and P4 for adolescents with AIS with right main curve; C3, Cp1 and P3 for adolescents with AIS with left main curve) and $P_C$ is the average of power of central electrodes contralateral to the curve (C3, Cp1 and P3 for right main curve; C4, Cp2 and P4 for left main curve). LI can thus range from + 1 (exclusively ipsilateral) to $-1$ (exclusively contralateral).

Considering that the curve may cause an asymmetric EEG topography, we a priori divided adolescents with AIS with right ($N= 10$ participants) and left ($N= 4$) main curve in performing the statistical comparisons with controls. Because relative power values were not all normally distributed (data distribution was tested by using Lilliefors test^28^), non-parametric tests were applied. We performed two-sided Wilcoxon rank sum tests to compare relative power in each frequency bands among controls and adolescents with AIS with right main curve ($p < 0.05$). Considering the sample size and the exploratory nature of the study, no corrections for multiple comparisons were performed in hypothesis testing.

### Balance and motion data

To test whether adolescents with AIS have an impaired balance control, we asked all the participants to perform the postural trials on a force platform, which returns the ground reactions and the Center of Pressure (COP) trajectory. COP is considered as a proxy of the Center of Mass movement and, thus, meaningful for balance performance^29^. Participants were instructed to find a comfortable position for their feet while standing on the force platform (9 components; BERTEC 4060-10, Bertec Corporation, Ohio, US; 2000 Hz). Feet position was marked on the floor with adhesive tape to ensure consistent positioning among trials. The relative foot placement was measured to estimate the base of support, which is approximated to the area of a trapezium having the following dimensions: the big toe distance (BTD) as the largest parallel side, the inter-malleolar distance (IMD) as the smallest parallel side, and the distance of big toes from the line joining the heel extremities (effective foot length, EFL) as the height^29^. Additionally, the maximum foot width (MFW) was collected, defined as the widest aspect of the foot, perpendicular to line joining the distal end of the great toe to midpoint of the heel^29^. Participants were asked to stand as still as possible to record the Center of Pressure (COP) trajectory and assess their balance control performances. Before calculating balance parameters, COP time series were low-pass filtered with a recursive 2nd-order Butterworth filter, cut-off equal to 10 Hz. Balance parameters might be affected by the duration of the acquisition. Thus, a single epoch of 40 s was retained for the analysis and the computation of the balance parameters from each of the recordings (lasting 3 min for the upright *standing* and 1 min for the *up arms* condition)^30^.

The following parameters were calculated from the COP trajectory to assess balance control performances of the participants: (1) length of COP trajectory (PL); (2) area of the ellipse containing the 95% of the COP points (EA); (3) range of motion (ROM) and root mean square (RMS) of the COP displacement along the anterior–posterior (ROM-AP and RMS-AP, respectively) and medial-lateral (ROM-ML and RMS-ML, respectively) directions^31^. Parameters were then normalized by the participants’ height, body mass, base of support and maximum foot width through a detrending normalization technique^29,32^. The detrending normalization consists in estimating the correlation between each parameter and subjects’ anthropometry, and then iteratively correcting each parameter with the linear model that best fits the data. This method keeps values with their original range and measurement unit, but it removes any dependencies from confounding variables^32^.

The postural task performed in the *OE arms up* and *CE arms up* conditions was expected to further challenge the balance of the adolescents with AIS, inducing a larger postural unbalance on the frontal plane, which is the plane most affected by the scoliotic deformity. Such a challenging position could affect the ability of adolescents with AIS to hold the arms’ position during the trial. To estimate the elbow angle $\varepsilon (t)$ in the *OE arms up* and *CE arms up* conditions, participants were equipped with nine retroreflective markers placed on the tubercle of the seventh cervical vertebra (C7), on the posterior aspect of the acromion (bilaterally and on the most prominent point), on the lateral epicondyle of humerus (bilaterally), and on both styloid process of ulna and radius (bilaterally). 3D marker trajectories were collected with a 10 IR-camera stereophotogrammetric system (Vero v2.2, Vicon Motion Systems Ltd, UK; 100 Hz). Labelling, gap filling and smoothing through a Woltring routine^33^ of marker trajectories were conducted within Vicon Nexus (v2.11, Vicon Motion Systems Ltd, UK).

In brief: the average of the wrist markers approximates the wrist joint centre, and the forearm segment is defined by the line joining the elbow marker to this point; the arm segment is defined by the line joining the shoulder marker to the elbow marker; the elbow angle time series $\varepsilon (t)$ is estimated as the angle between the forearm and the arm segments. The following parameters have been defined to quantify the variation over time of the elbow angle and its symmetry between left and right sides:

- directional root mean square (dRMS, $^\circ$):
  2 $$\begin{aligned} \begin{aligned} dRMS = \Biggl \{ \begin{matrix} RMS\{\varepsilon (t)\},~~\text {if}~~{\bar{\varepsilon }}(t)|_{t = 38, \dots , 40}-{\bar{\varepsilon }}(t)|_{t = 1, \dots , 3}\ge 0\\ -RMS\{\varepsilon (t)\},~~\text {if}~~{\bar{\varepsilon }}(t)|_{t = 38, \dots , 40}-{\bar{\varepsilon }}(t)|_{t = 1, \dots , 3} < 0\\ \end{matrix} \end{aligned} \end{aligned}$$
  with ${\bar{\varepsilon }}$, being the time average of the elbow angle over the selected time window;
- coefficient of variation of the angle (CV, %), i.e., the $\frac{RMS}{{\bar{\varepsilon }}_{(t)}}\cdot 100$;
- Symmetry index (SI, %):
  3 $$\begin{aligned} \begin{aligned} SI = \frac{{\bar{\varepsilon }}_L(t)-{\bar{\varepsilon }}_R(t)}{{\bar{\varepsilon }}_L(t)+{\bar{\varepsilon }}_R(t)}\cdot 100. \end{aligned} \end{aligned}$$

Balance and motion data were tested for possible differences between-groups (AIS vs. CTRL) through an unpaired two-sided Wilcoxon rank sum tests ($p < 0.05$). Differences in balance performances due to the eye condition (*OE* vs *CE* comparison) were tested through a paired two-sided Wilcoxon rank sum test ($p <0.05$), to account for repeated measures design within the same group (AIS and CTRL).

### Body schema assessment

A re-adaptation of the Image Marking Procedure (IMP) was used to assess the body schema^25^. The IMP was previously used to assess body schema, especially body size perception (horizontal direction) and general body awareness^34^. This projection test assesses subjects’ ability to implicitly represent their body (specifically those parts more affected by scoliosis: shoulder, back, waist) based on a tactile stimulation. As it was never used before on adolescents with AIS, we re-adapted it to evaluate both the horizontal and vertical body representation. Participants were asked to stand blindfolded in front of a roll of wrapping paper, pretending looking themselves in a mirror. The experimenter stood behind the subject firmly touching with his fingertip seven chosen body segments, namely: the top of the head, the acromioclavicular joints (right and left), waist width (right and left), and the trochanters of the femoral bones (right and left). Each participant was provided with a pencil and asked to make a cross on the paper where the touched body segment was imagined to be projected (see Supplementary Materials). At the end of the marking, participants approached the paper to allow the experimenter to mark the actual position of the touched points with the use of an L-shape ruler placed at 90$^{\circ }$ angle relative to the longitudinal axis of the body. Distances in cm between the corresponding right and left points of shoulders, waist and hips marked by the subjects (perceived size: PS) and by the experimenter (actual size: AS) were measured with a ruler. For each subject, body perception indices (*BPIs*) related to each body segment (shoulders, waist, and hips) were calculated using the following formula:

4 $$\begin{aligned} \begin{aligned} BPI = \frac{PS}{AS}*100 \end{aligned} \end{aligned}$$

A general *BPI* (*BPIg*), determined as the *BPIs* average was calculated as well. The classification of *BPIs* was performed through normative values^35^: hypo-schematic (underestimation of actual body size, $BPIg < 99.4\%$; adequate body size estimators ($BPIg > 99.4\%$); hyper-schematic (over-estimation of actual body size, $BPI > 112.3\%$).

To assess possible body schema distortions on the vertical axis, we compared the actual deviation from verticality (degrees) and its lateralization (right or left) with the perceived ones. Toward this aim, we defined for each subject an angle formed by the prolongation of the segments connecting right and left perceived points of shoulders, waist or hips, (depending on the principal curve location), and by the segment connecting the corresponding right and left actual points. To characterize curve laterality, we assumed positive values if the perceived curve convexity was right-oriented, and negative values if it was left-oriented (see Supplementary Materials).

As *BPIs* scores were normally distributed (Shapiro–Wilk normality test) we performed unpaired two-sampled t-test to compare AIS and controls *BPIs* scores related to each body segment and *BPIs* average scores ($p < 0.05$). To evaluate eventual body schema distortions related to the whole trunk perception, we additionally calculated *BPIs* shoulder/waist ratio, as the delta between these two measures and we performed unpaired two-sample t-test between groups ($p < 0.05$).

All methods were performed in accordance with the relevant guidelines and regulations.

### Ethics approval and consent to participate

Informed consent was given from all subjects and/or their legal guardian(s) for participation and publication of identifying information/images in this study. All methods were performed in accordance with relevant guidelines and regulations and in accordance with the Declaration of Helsinki. This study was carried out in accordance with the recommendations of the Ethics Committee of the Teaching Hospital of Padova.

## Results

### EEG data

**Figure 1:**
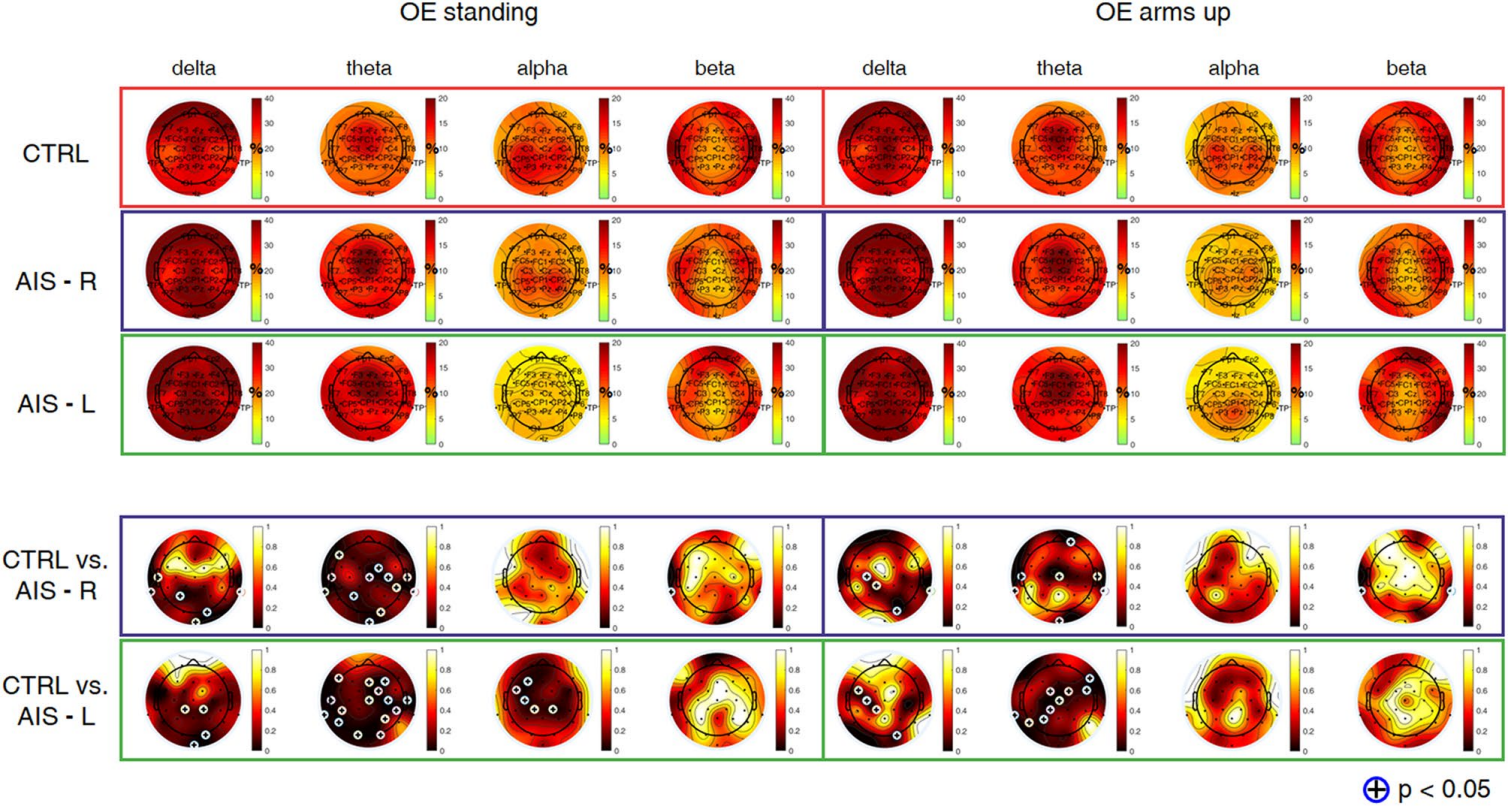
Topographic maps of relative power (%) in delta [1–4] Hz, theta [4.5–7.5] Hz, alpha [8–12] Hz, and beta [13–30] Hz bands, during *OE standing* and *OE arms up*, averaged from 14 controls (CTRL) (first row), 10 adolescents with AIS with right main curve (second row) and 4 adolescents with AIS with left main curve (third row). The fourth and the fifth rows represent *p*-maps derived from the Wilcoxon rank sum test (adolescents with AIS with right main curve: AIS-R vs. CTRL; adolescents with AIS with left main curve: AIS-L vs. CTRL). Statistical results were highlighted with (+) for $\hbox {p} < 0.05$.

**Figure 2:**
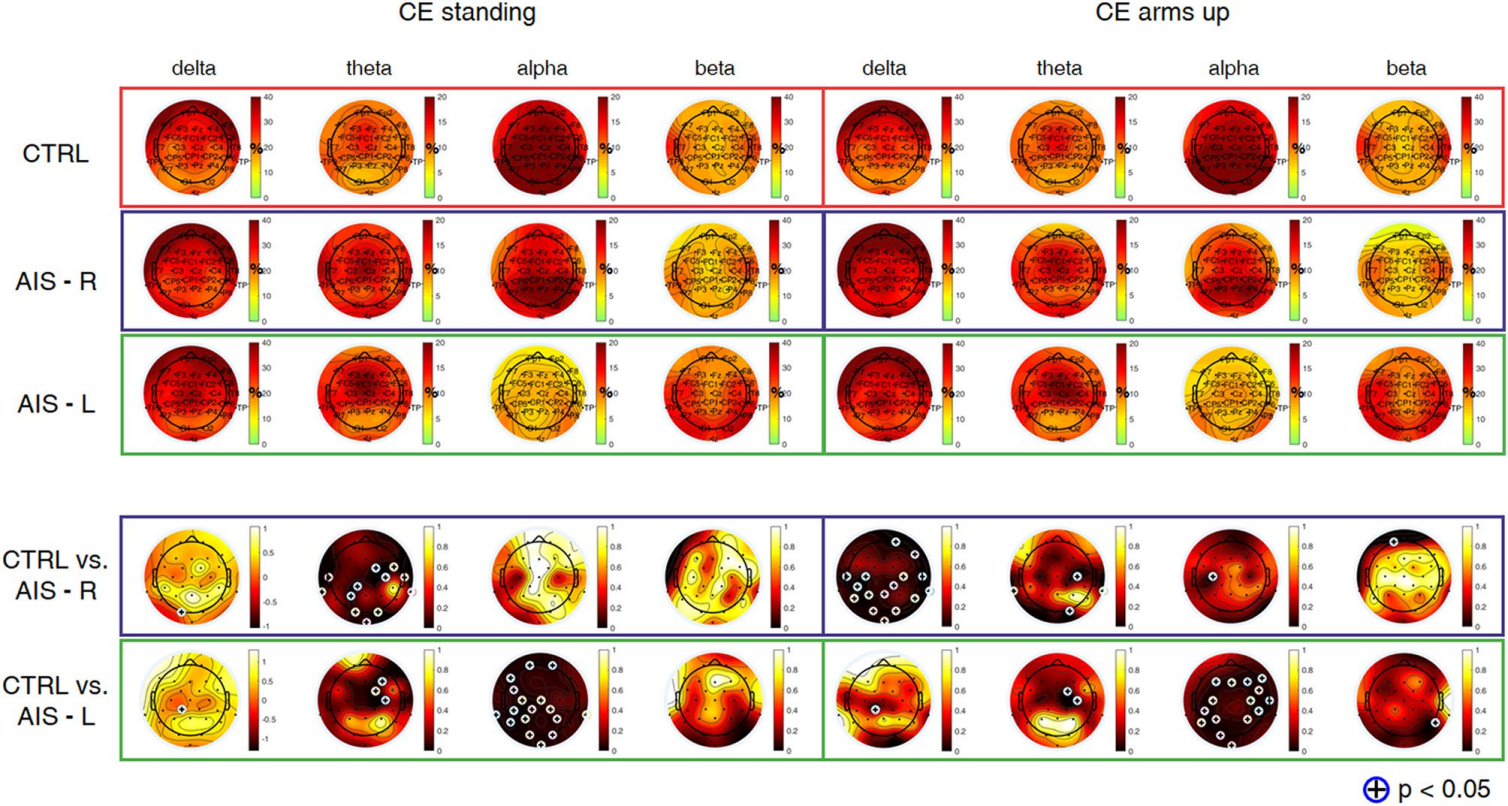
Topographic maps of relative power (%) in delta [1–4] Hz, theta [4.5–7.5] Hz, alpha [8–12] Hz, and beta [13–30] Hz bands, during *CE standing* and *CE arms up*, averaged from 14 controls (CTRL) (first row), 10 adolescents with AIS with right main curve (second row) and 4 adolescents with AIS with left main curve (third row). The fourth and the fifth rows represent *p*-maps derived from the Wilcoxon rank sum test (adolescents with AIS with right main curve: AIS-R vs. CTRL; adolescents with AIS with left main curve: AIS-L vs. CTRL). Statistical results were highlighted with (+) for $\hbox {p} < 0.05$.

Average results from power spectral density analysis are summarized in Figs. 1 and 2 for controls and adolescents with AIS. In the *OE condition* (Fig. 1), significant differences were observed during *OE standing* and *OE arms up* between controls and adolescents with AIS with right and left main curves in slow rhythms (i.e., delta and theta) over central and parietal electrodes ($p =0.0417$). Comparison between controls and adolescents with AIS with left main curve indicated significant differences in alpha range also over left sensorimotor and parietal areas during *OE standing* ($p =0.0415$).

In the *CE condition* (Fig. 2), significant differences were observed during *CE standing* between controls and adolescents with AIS with right main curve in theta rhythm over central and parietal electrodes ($p =0.0218$). *CE arms up* produced significant differences in delta band over central and parietal electrodes ($p =0.0225$) and in alpha band over C3 ($p =0.0326$). Comparison between controls and adolescents with AIS with left main curve indicated significant differences over central regions in delta and theta bands during both tasks ($p =0.0384$) and over left sensorimotor and parietal areas during *CE standing* ($p =0.0354$) and over both sensorimotor areas in alpha band during *CE arms up* ($p =0.0340$). Relative powers were comparable between the two postures within each condition (*OE* and *CE*).

**Figure 3:**
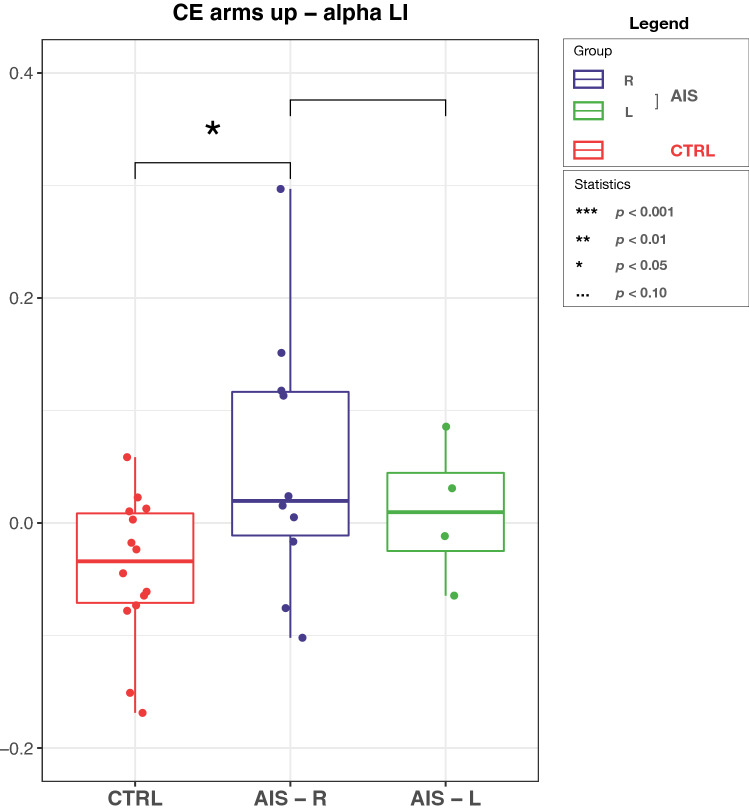
Grand-average LI in alpha band during *CE arms up* for controls (CTRL), adolescents with AIS with right main curve (AIS-R) and adolescents with AIS with left main curve (AIS-L). On each box, the central red line indicates the median, and the bottom and top edges of the box indicate the 25th and 75th percentiles, respectively. The whiskers extend to the most extreme data points not considered outliers, and the outliers are plotted individually using the ‘+’ symbol. Significant difference ($\hbox {p} < 0.05$) was observed between CTRL and AIS-R.

EEG data demonstrated alpha power increase of the sensorimotor area ipsilateral to the main scoliotic curve, as evidenced by the significant changes of LI ($p =0.0434$) mainly in adolescents with AIS with right main curve during *CE arms up* (Fig. 3).

### Balance and motion data

**Figure 4:**
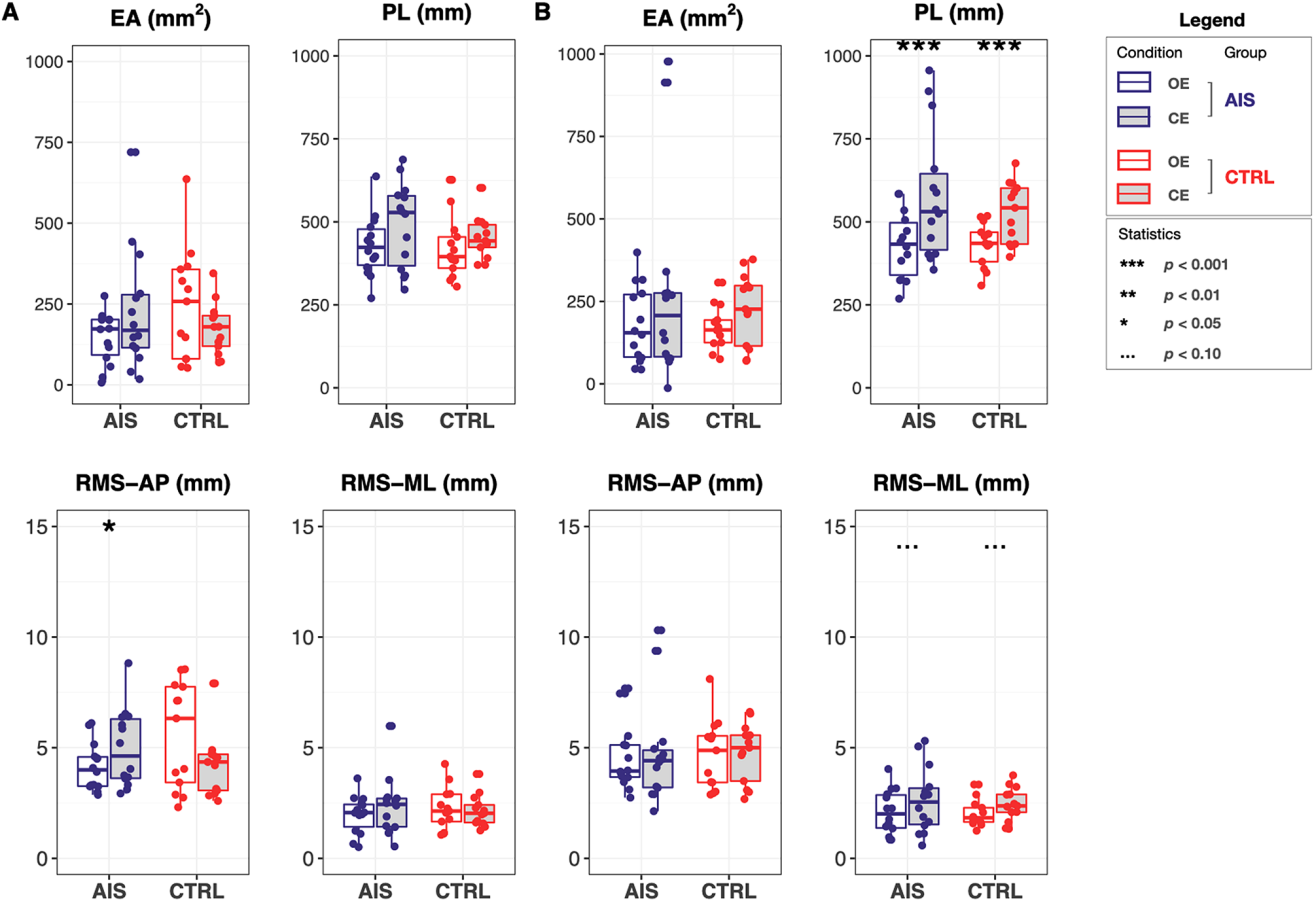
Balance performances as assessed through confidence ellipse area (EA), sway path length (PL) and the root mean square of center of pressure trajectory in the anterior–posterior and medial-lateral directions (RMS-AP and RMS-ML, respectively) in: (**A**) *standing*, and (**B**) *arms up* conditions. Boxplots are filled with white for eyes open (*OE*) and gray for eyes closed (*CE*). Statistical results were highlighted with: ‘***’ for $p<0.001$, ‘**’ for $p<0.01$, ‘*’ for $p<0.05$, and ‘...’ for $p<0.10$.

Figure 4 shows results of balance performances as assessed through EA, PL, RMS-AP and RMS-ML in *standing* (panel A) and *arms up* (panel B) conditions with both eyes open and closed (white and gray fill color, respectively) in both adolescents with AIS and controls. No differences were obtained between groups, whereas a strong significance (AIS: $p =0.00085$; CTRL: $p =0.00049$) was obtained when testing differences between *OE arms up* and *CE arms up* conditions for PL (Fig. 4B). A weak significant difference ($p =0.035$) was obtained for RMS-AP in adolescents with AIS between *OE* and *CE standing* (Fig. 4A).

From results on motion data, the only significant difference was found for the SI in the controls ($p<0.013$) (See Supplementary Materials).

### Body schema data

*BPIg* scores did not differ between controls and adolescents with AIS (AIS: mean ± SD $=88.47 \pm 19.75$; CTRL: $86.31 \pm 16.64$; $p = 0.75$) as well as *BPIs* related to each body segment: *BPI* of shoulders (AIS: $81.88 \pm 21.24$; CTRL: $85.55 \pm 18.00$; $p = 0.62$), *BPI* of waist (AIS: $97.94 \pm 31.13$; CTRL: $91.37 \pm 16.03$; $p = 0.48$), *BPI* of hips (AIS: $85.59 \pm 21.39$; CTRL: $82.00 \pm 22.75$; $p = 0.67$). The *BPIg* scores in both controls and adolescents with AIS, resulted mainly hypo-schematic with 8 of 14 adolescents with AIS and 12 of 14 controls having *BPIg* scores lower than $99.4\%$.

**Figure 5:**
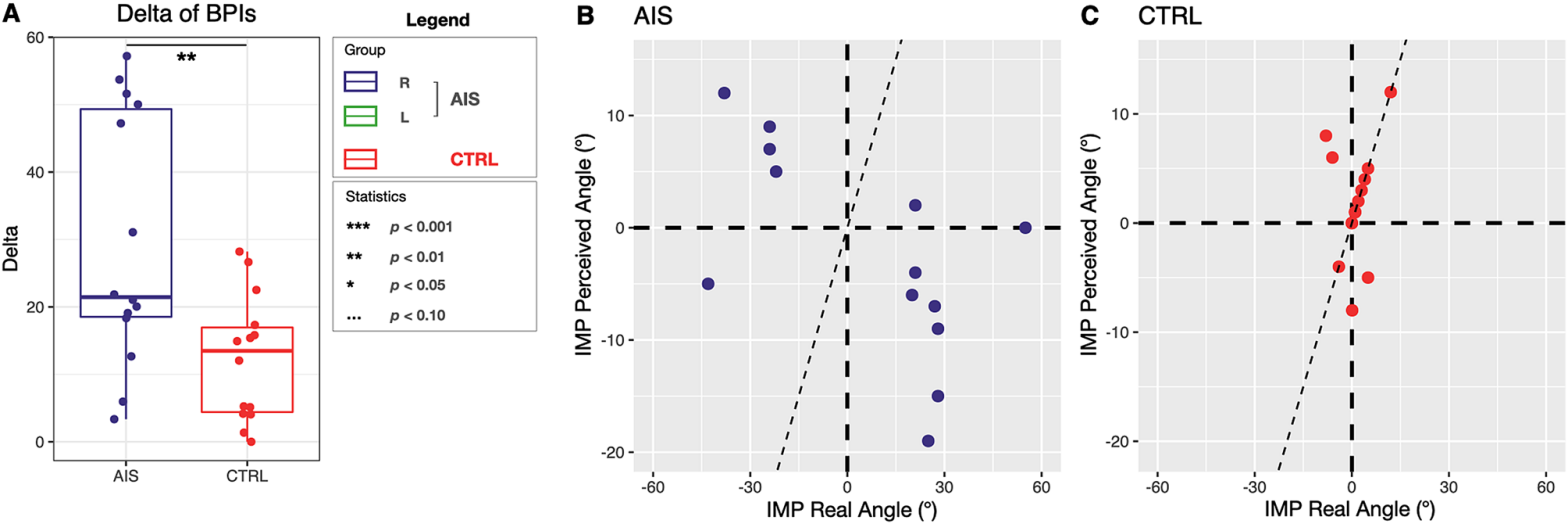
(**A**) *BPI* delta values defined as the difference between shoulder *BPI* and waist *BPI*. A significant difference emerged between mean delta *BPIs* of adolescents with AIS and controls ($p < 0.01$). (**B,C)**) On the x axis are plotted the real angles as measured with IMP while on the y axis the perceived angles as measured with IMP. To characterize curve laterality, we assigned positive values when curves’ convexity was right-oriented, and negative values when left-oriented. (**B**) In the adolescents with AIS 12 out of the 14 girls perceive their curve orientation as opposite to the real one. (**C**) For the control group, just 3 out of 14 girls perceive their curve orientation as opposite to the real one.

The *BPI* delta (*BPI* shoulder minus *BPI* waist) resulted significantly different between the two groups (mean ± SD *BPI* delta of AIS group: 29.52 ± 18.72 vs. controls: 12.35 ± 9.3; $p=0.004$) (Fig. 5A).

Qualitative analysis of the test showed that in 12 out of 14 adolescents with AIS the inclinations of the perceived interacromial axes in girls having thoracic principal curve and bisiliac axes in girls with lumbar principal curve was opposite to the real inclination (Fig. 5B). Conversely, controls’ perception of their inclination resulted opposite to the real inclination in 3 out of 14 cases (Fig. 5C). The real angle (i.e., degrees describing the inclination of the interacromial or bisiliac axes resulting from the actual projection of body segments, as measured by the experimenter) and the perceived angle (i.e., degrees describing the inclination of the interacromial or bisiliac axes resulting from the participants’ perceived projection of body segments) should coincide in a healthy subject and be represented by the points lying over the bisector of the first and third quadrant in Fig. 5B,C.

## Discussion

The three aims of this exploratory study were: (1) to compare the EEG activity in adolescents with AIS and controls during posture; (2) to unveil possible alterations in the postural control mechanisms and (3) to investigate body schema alterations. We hypothesized that adolescents with AIS compared to controls might exhibit: (1) altered brain activation of the sensorimotor network; (2) larger sway on the frontal plane, i.e., the plane with the larger scoliotic deformity; (3) altered body schema reflecting the altered activation of the sensorimotor network. Our results revealed: (1) significant increase of delta and theta relative powers, compared to controls, localized over the central areas; a significant LI increase in alpha range observed in adolescents with AIS with main right curve compared to controls during *arms up* with eyes closed, demonstrating an imbalance between the two sensorimotor areas; (2) no differences in balance performances between groups; (3) no differences between controls and adolescents with AIS for the single body segment, but the test of body schema underlined a possible alteration in the overall trunk representation, namely in the perception of the shoulders-waist proportion, and in its inclination.

The main result of the study is the significant increase of delta and theta relative powers, compared to controls, localized over the central areas. Theta frequency band is recognized to be involved in balance control^36^. Theta band activity intensifies when control demands are increased^37^; it is reported to rise with increasing balance demand in parietal areas^38^, as well as in frontal areas^39–41^; it has also been suggested to represent error detection and processing during postural maintenance^38^. Theta power increase with task complexity has been demonstrated in multiple contexts, including dual tasks^36^ where an increase of cortical recruitment was found to keep up performance in the elderly.

Gebel et al.^42^ found significant increases of theta frequency band power in frontal and central areas with increasing balance task difficulty in adolescents. Moreover, previous studies on healthy adults reported that the increase in theta power may originate from the anterior cingulate cortex and sensorimotor areas, which are highly involved in sensory information processing^38,43^.

In adolescents with AIS, the theta power increase during all the tasks within central clusters may reflect a higher information processing load due to increased postural demands caused by the scoliosis. The no balance difference and the increased brain activity in this frequency range could reflect greater postural control during balance tasks (with eyes open and closed, with and without arms up). These results (i.e., the overall theta increase over central areas) suggest that adolescents with AIS adapted their brain activity to prevent large body sway due to the scoliosis.

The lateralization of alpha relative power is in line with fMRI data showing Blood Oxygen Level Dependent (BOLD) activation increase of motor areas and a greater interhemispheric asymmetry index^14^. The overactivation of contralateral supplementary motor area, observed when performing motor task with either hand, emphasizes the asymmetry of motor activation and demonstrates an abnormal pattern of brain activation supporting the hypothesis of sensorimotor dysfunction. The increase of alpha power lateralization could indicate an increase of communication relayed to the sensorimotor area ipsilateral to the scoliotic curve due to the balance task. The alpha lateralization observed in our results may be a compensatory strategy to overcome sensorimotor dysfunction. A possible explanation of these changes could be attributed to impaired sensorimotor integration predominantly at the cortical level, results found also in other studies^14,16^. Brain oscillations, thanks to neural plasticity, can reorganize in a system attempting to compensate for a dysfunction; the asymmetry observed in adolescents with AIS may be related to either a sensorimotor impairment or a brain compensatory mechanism. Maybe scoliosis onset is preceded by sensorimotor control impairments that last during curve progression. This in turn could lead to develop an altered body schema of the trunk and its inclination. We infered that the “unbalance” in alpha power spectra between the two hemispheres in the sensorimotor areas might be linked with the “unbalance” in body perception in AIS. Indeed, we evidenced: (1) an alpha lateralization ipsilateral to the scoliotic curve; (2) the opposite inclination of the perceived interacromial axes compared to the real inclination; and (3) an abnormally widest perceived waist compared to shoulders only in adolescents with AIS. Further investigations are worth performing to investigate this relationship. However, we cannot exclude the possibility that our finding on the abnormally widest perceived waist compared to shoulders could be the result of brace treatment. Body schema is plastic by definition, and capable of incorporating objects in short times^44^.

Before identifying the therapeutic target, other neurophysiological investigations must be carried out: high-density EEG to localize cortical sources; connectivity analysis to understand whether the alterations detected in the rhythms are also reflected in terms of connections between the brain areas of the motor sensory network. A methodological limitation of this study is the approach of sensor space localization by means of only 30 EEG channels. Source localization analysis requires at least an EEG system with 64 channels for data acquisition. Therefore, future studies may use high-density EEG systems to specify and localize the functional areas during increasing postural demands. Moreover, it could be interesting to explore the influence of brace on body perception. In this pilot study, indeed, we only considered participants which already underwent brace treatment. Another limitation is the limited number of participants. We faced some issues in recruitment since recordings were performed during the second lock-down in Italy due to COVID-19 pandemic (i.e., Veneto region barred residents from leaving their homes except for work, health or basic needs, and among commercial activities, only supermarkets and pharmacies stayed open).

The obtained results lead to refuting the initial hypothesis of adolescents with AIS having worse balance performance than controls, even when considering the postural task with the arms up that we expected to unveil and magnify such differences between the two groups. However, when looking at the symmetry index (SI) calculated on the elbow angle in the up arms condition, controls did act differently in the eyes open and eyes closed condition ($p = 0.013$), whereas no differences were obtained within the adolescents with AIS. This different behavior is probably to be ascribed to the larger variability that characterizes the SI values calculated for adolescents with AIS, compared to controls, which may again suggest different postural control of the adolescents with AIS. Building on this consideration, we would expect to observe different brain cortical activity on the sensorimotor areas between adolescents with AIS and controls, which we indeed observed on EEG rhythms associated with postural control. Although further investigations are worth performing, we may conclude that adolescents with AIS have no functional balance disorder, but they show different and asymmetrical activation of the sensorimotor areas of the brain (i.e., theta increase and alpha lateralization). Theta increase might represent a higher level of attention^36^ in performing symmetric tasks in adolescents with AIS and alpha lateralization might stand for the brain reorganization in a system attempting to compensate for a dysfunction. This dysfunction is testified by the altered body schema (i.e., opposite to the real one) in adolescents with AIS.

A longitudinal study with a larger cohort is still needed to verify if these abnormal EEG findings may represent a valuable biomarker of scoliosis progression. The results can offer novel therapeutic targets as for example training based on biofeedback, evaluating the performance by monitoring EEG changes. The marked lateralisation observed and body schema alterations could also be useful for setting up personalized and targeted corrective postural exercises. Nowadays, treatment options for scoliosis consider exclusively biomechanics perspectives, except for some innovative approaches and hypotheses, still needing scientific evidence. One future development is the investigation of the EEG as a tool to assess the probable development of scoliosis in the individual case. Thanks also to the development of mobile EEG systems, it will be possible to study the sensorimotor components even during movement tasks used to increase postural instability (e.g. by using a balance board). It still remains unclear whether the reported cortical functional state changes are either the cause or consequence of AIS. A longitudinal study is needed to find evidence to try to clarify this aspect.

## Conclusions

Our results provide evidence of an increased theta activity and a lateralized alpha activity in adolescents with AIS. Whether these processes are a cause or a consequence of AIS needs to be further investigated. The identification of an abnormal EEG pattern may describe a much more complex physiopathology of AIS and promote new multi-domain treatment approaches to improve patients’ care quality.

## Supplementary Information

Supplementary Information.

## Data Availability

The datasets generated and/or analysed during the current study are not publicly available due to participants’ privacy. Anonymous Data and Code will be made available upon reasonable request to the Corresponding Author. None of the experiments was preregistered.

## Abbreviations

AIS: Adolescent idiopathic scoliosis
CNS: Central nervous system
PPC: Posterior parietal cortex
MRI: Magnetic resonance imaging
fMRI: Functional magnetic resonance imaging
EEG: Electroencephalography
TMS: Transcranial magnetic resonance imaging
LI: Laterality Index
BTD: Big toe distance
IMD: Inter-malleolar distance
EFL: Effective foot length
MFW: Maximum foot width
COP: Center of preassure
PL: Sway path length
EA: Ellipse area
AP: Antero-posterior
ML: Medio-lateral
RMS: Root mean square
SI: Simmetry index
QoL: Quality of life
SRS: Scoliosis Research Society
PS: Perceived size
AS: Actual size
*BPI*: Body Perception Index
*BPIg*: General Body Perception Index
