# Supplementary material for: Brain oscillatory activity in adolescent idiopathic scoliosis
Source: Sci Rep. 2022 Oct 14;12:17266. doi: 10.1038/s41598-022-19449-1 (PMC9568615; doi:10.1038/s41598-022-19449-1)
Supplement: Supplementary file 1 — Supplementary Information. [file 41598_2022_19449_MOESM1_ESM.pdf]

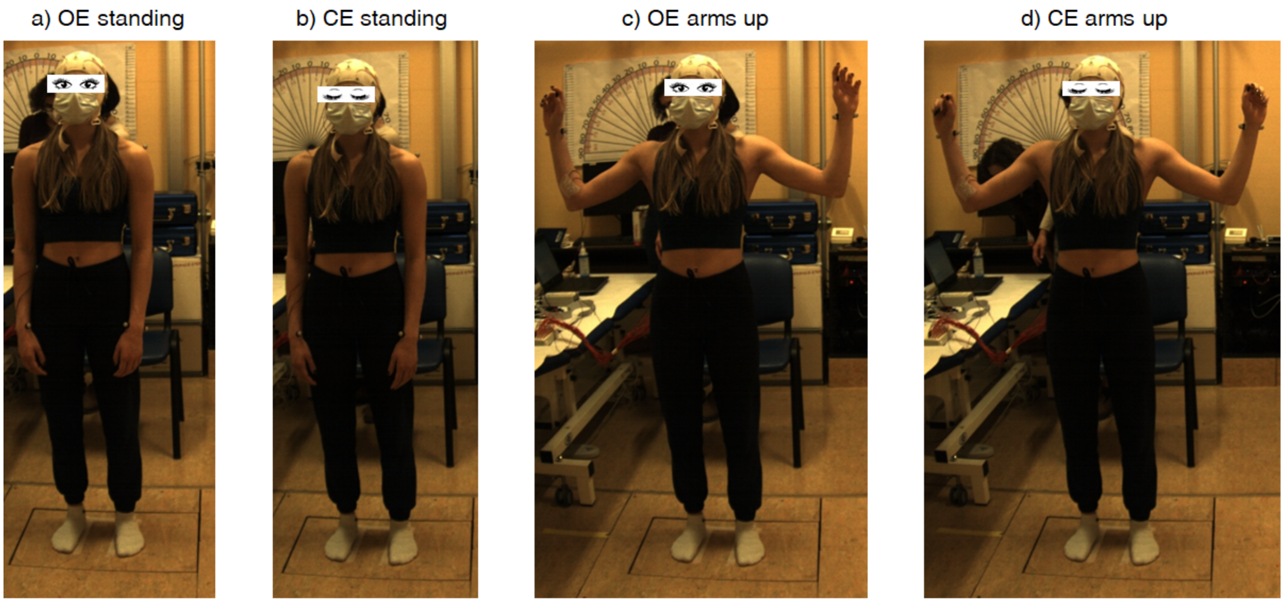

Supplementary Figure S1. *Experimental Paradigm.* Data were acquired in different upright standing postures (on a force platform): with eyes open (a) and closed (b); with arms raised to 90 degrees with eyes open (c) and closed (d).

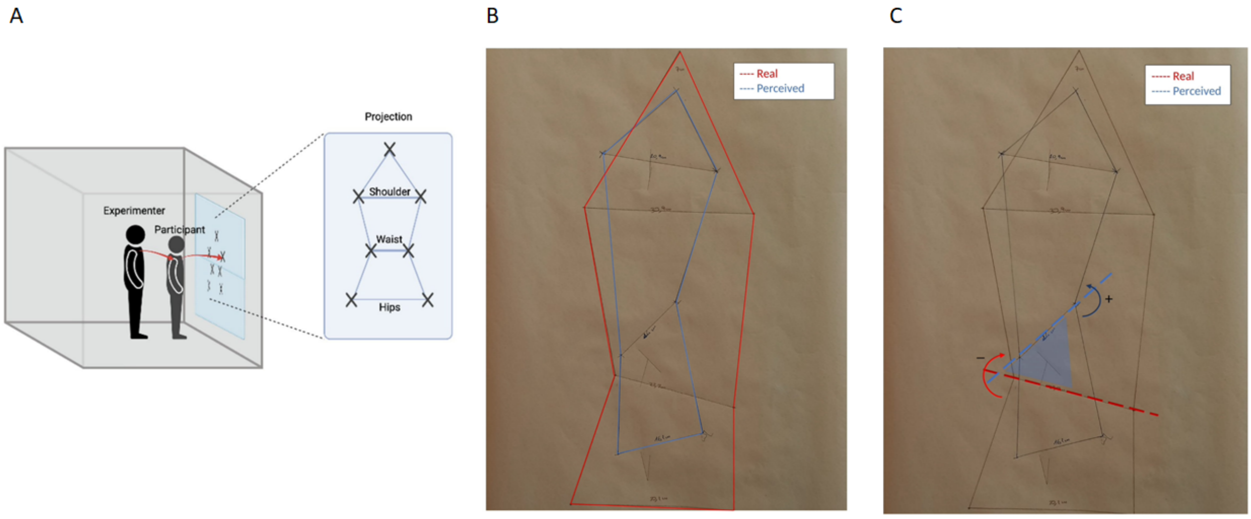

Supplementary Figure S2. *Graphic representation of the IMP setting, performance, and angles computation.* Representation of the IMP setting (A)*. Example of IMP performed by a girl of the AIS group (B). The red lines represent the real distances between the touched points, as measured by the experimenter with an L-shape ruler. The blue lines represent the perceived projection of the touched points, as indicated by the girl. The girl in the reported example had a principal right oriented thoraco-lumbar curve (25° Cobb). We considered the angle formed prolonging the segment connecting the perceived waist points (blue line) and the one connecting the real waist points (red line). We attributed positive values to the angles whose convexity was right oriented and negative values to left oriented ones (C).

*This image was drawn by Margherita Bertuccelli.

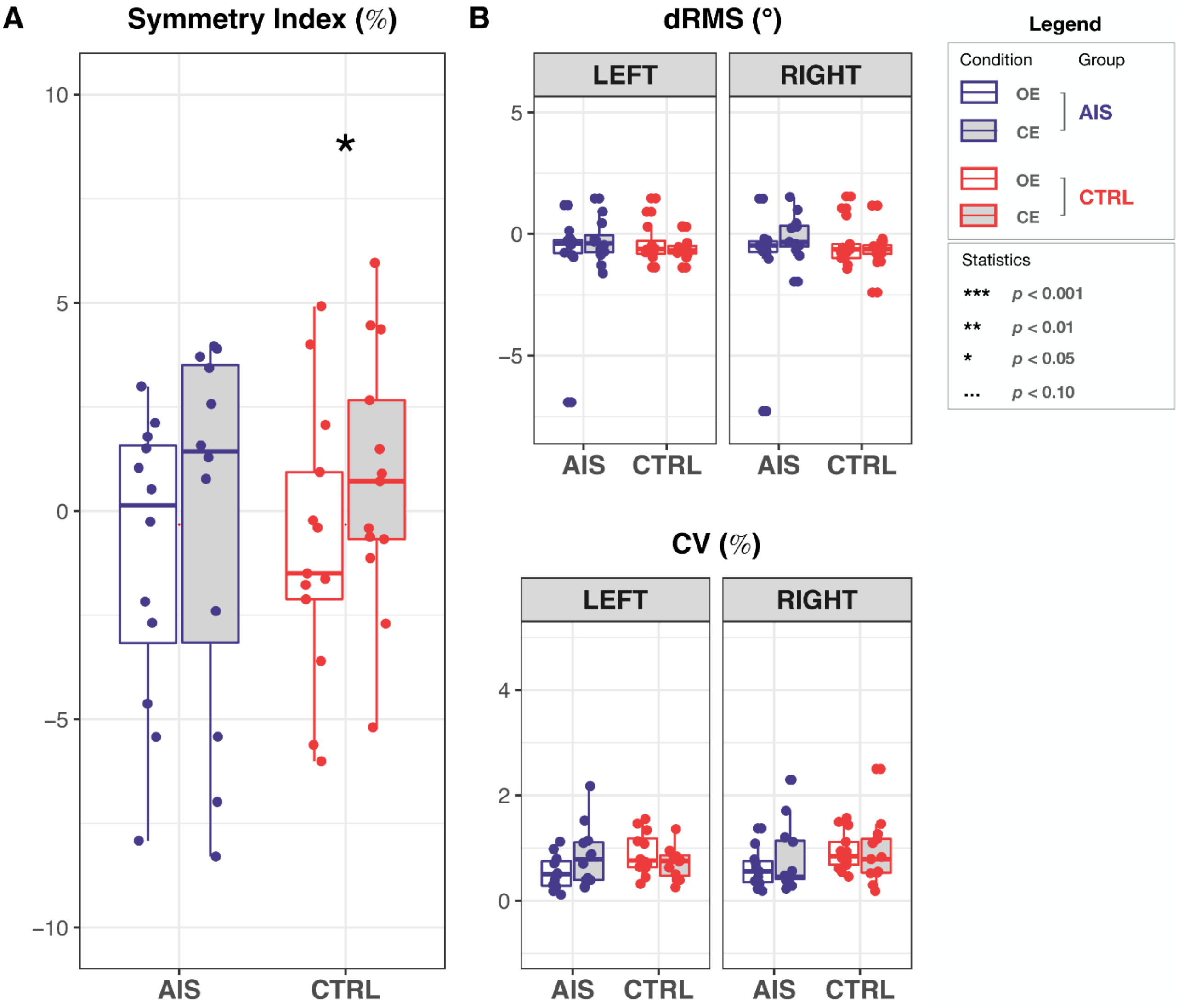

Supplementary Figure S3. *Results on motion data in arms up posture.* The figure shows the results obtained for motion data. In particular, panel A shows the results for the symmetry index (SI), calculated for conditions OE and CE arms up. Significant differences were obtained for the control group when looking at this index (p<0.05), whereas no differences were obtained for the AIS group. Panel B, instead, shows both the directional root mean square (dRMS) and coefficient of variation of the elbow angle in both groups and both OE and CE arms up conditions for left and right upper limbs.
